# Exposure to Morphine and Caffeine Induces Apoptosis and Mitochondrial Dysfunction in a Neonatal Rat Brain

**DOI:** 10.3389/fped.2020.00593

**Published:** 2020-09-18

**Authors:** Sweatha Kasala, Seema Briyal, Preetha Prazad, Amaresh K. Ranjan, Gospodin Stefanov, Ramona Donovan, Anil Gulati

**Affiliations:** ^1^Division of Neonatology, Department of Pediatrics, Advocate Children's Hospital, Park Ridge, IL, United States; ^2^Chicago College of Pharmacy, Midwestern University, Downers Grove, IL, United States; ^3^Advocate Aurora Research Institute, Park Ridge, IL, United States; ^4^Pharmazz Inc. Research and Development, Willowbrook, IL, United States

**Keywords:** morphine, caffeine, mitochondrial function, apoptosis, neonatal rat model, brain

## Abstract

**Background:** Preterm infants experience rapid brain growth during early post-natal life making them vulnerable to drugs acting on central nervous system. Morphine is administered to premature neonates for pain control and caffeine for apnea of prematurity. Simultaneous use of morphine and caffeine is common in the neonatal intensive care unit. Prior studies have shown acute neurotoxicity with this combination, however, little information is available on the mechanisms mediating the neurotoxic effects. The objective of this study was to determine the effects of morphine and caffeine, independently and in combination on mitochondrial dysfunction (Drp1 and Mfn2), neural apoptosis (Bcl-2, Bax, and cell damage) and endothelin (ET) receptors (ET_A_ and ET_B_) in neonatal rat brain.

**Methods:** Male and female rat pups were grouped separately and were divided into four different subgroups on the basis of treatments–saline (Control), morphine (MOR), caffeine (CAFF), and morphine + caffeine (M+C) treatment. Pups in MOR group were injected with 2 mg/kg morphine, CAFF group received 100 mg/kg caffeine, and M+C group received both morphine (2 mg/kg) and caffeine (100 mg/kg), subcutaneously on postnatal days (PND) 3–6. Pups were euthanized at PND 7, 14, or 28. Brains were isolated and analyzed for mitochondrial dysfunction, apoptosis markers, cell damage, and ET receptor expression via immunofluorescence and western blot analyses.

**Results:** M+C showed a significantly higher expression of Bax compared to CAFF or MOR alone at PND 7, 14, 28 in female pups (*p* < 0.05) and at PND 7, 14 in male pups (*p* < 0.05). Significantly (*p* < 0.05) increased expression of Drp1, Bax, and suppressed expression of Mfn2, Bcl-2 at PND 7, 14, 28 in all the treatment groups compared to the control was observed in both genders. No significant difference in the expression of ET_A_ and ET_B_ receptors in male or female pups was seen at PND 7, 14, and 28.

**Conclusion:** Concurrent use of morphine and caffeine during the first week of life increases apoptosis and cell damage in the developing brain compared to individual use of caffeine and morphine.

## Introduction

Preterm neonates in the Neonatal Intensive Care Unit (NICU) are subjected to many essential but painful procedures (1). A growing focus on the comfort and pain control in the NICU has led to an exponential increase in opioid administration (2). Although opioid management for acute pain is thought to be evidence-based, prolonged use of opioids in neonates remains a concern secondary to its potential deleterious effects on the developing brain (3, 4). Caffeine citrate is a standard treatment for apnea of prematurity (AOP) in the extremely premature infants. The “Caffeine for Apnea of Prematurity (CAP)” trial demonstrated safety, tolerability, and efficacy of caffeine for treatment of apnea, with reduction in bronchopulmonary dysplasia (BPD) and improvement in survival of 18–21 month old preterm infants without neurodisability (5). Despite the widespread clinical use, effects of caffeine on the developing brain remained controversial. Morphine in association with caffeine is routinely used in the NICU (6, 7); however, there are conflicting reports surrounding morphine analgesia and its long-term neurologic effects in preclinical and clinical studies. Various *in vitro* (8, 9) and *in vivo* studies (10–12) have shown induced neuroapoptosis because of morphine treatment. Chronic morphine exposure has shown to cause a significant reduction in brain volume and dendritic growth (13), modification in synaptic neuroplasticity in limbic system and impairment in learning throughout adult life (14–16). It has also known to result in long-lasting neurochemical changes and affects hippocampal development (17, 18). Results from these studies suggest that morphine has neurotoxic effects; however, Zhaleh et al. has shown that use of a low-dose morphine could have suppressive effects on cytotoxicity (19). Moreover, a clinical study has shown an association between neonatal low-dose morphine analgesia and early alterations in cerebral structure as well as short-term neurobehavioral problems that did not persist into childhood (20). Conversely, several randomized trials have reported that short term or long-term morphine treatment in preterm neonates did not alter cognitive ability or motor development when measured at 5 and at 8–9 years of age (21–24).

Limited research exists on the safety and long-term consequences of caffeine on the developing brain. *In vivo* and *in vitro* studies have shown caffeine induced neuroapoptosis (25), alteration of astrogenesis (26), and transient motor impairment (27). Preclinical studies have shown that therapeutic doses of caffeine may significantly augment the neurotoxicity of sedative/anesthetic drugs (28), challenging the clinical assumption that caffeine is safe for premature infants when used in combination with sedative drugs.

Studies at the molecular level show that the developing rat brain from PND 1 to PND 7, closely correlates with the timing of synaptogenesis which is highly susceptible to neuroapoptosis (29, 30). The opioid and endothelin system modulate neuronal migration, differentiation and maturation during this period (31–34). Endothelin A (ET_A_) and B (ET_B_) receptors have been shown to play an important role in mitochondrial function in an adult stroke model. Blockage of ET_B_ receptors and activation of ET_A_ receptors appears to trigger apoptotic processes by modulating mitochondrial function (34). However, the involvement of ET receptors in relation to mitochondrial function in an animal model of drug-induced neurotoxicity is of interest and remained elusive.

Optimal mitochondrial function is crucial for brain development and function, including regulation of neurogenesis, neural stem cell differentiation (35, 36), and development of synapses (37, 38) which is controlled by mitochondrial fusion and fission (39). An abnormal increase in fission through dynamin-related protein 1 (Drp1), leads to mitochondrial insertion of pro-apoptotic proteins such as Bcl-2 associated × protein (Bax) triggering apoptosis. Anti- apoptotic proteins like Bcl-2 bind to Bax to prevent apoptosis, the balance between pro-apoptotic, and anti-apoptotic proteins on the mitochondrial membrane determine cell fate (40). Bcl-2 and Bax are present permanently on the mitochondrial and endoplasmic reticulum membranes controlling drug-induced mitochondria mediated apoptosis by regulating the calcium storage (41). Overexpression of mitofusin-2 (Mfn2) results in suppression of mitochondrial fragmentation leading to decreased apoptosis (42–44). Disturbance in fine balance between fusion/fission results in disturbed mitochondrial morphogenesis leading to altered development and function of immature synapses of nerves (37).

Since mitochondria may be the initial and one of the most vulnerable targets of drug-induced impairment of neuronal development, we hypothesized that caffeine in association with morphine would augment neuroapoptosis involving the activation of a mitochondria-dependent apoptotic cascade. This would manifest as an increase in the expression of ET_A_, Drp1, Bax, cell damage, and decrease in the expression of ET_B_, Mfn2, and Bcl-2. We also hypothesized that the central nervous system (CNS) protein expression would demonstrate gender-specific differences suggestive of female neuroprotective properties. The goal of the present study was to use a neonatal rat model to mimic clinical exposure to caffeine and morphine in the premature neonates and their effects on different stages of neurodevelopment through the specific CNS protein analyses.

## Materials and Methods

### Animals

Twenty timed-pregnant Sprague–Dawley rats (Envigo, Indianapolis, IN) were housed in a room with controlled temperature (23 ± 1°C), humidity (50 ± 10%), and light (6:00 A.M−6:00 P.M). All animals were maintained on a 12-h light/dark schedule. Food and water were available *ad libitum*.

### Experimental Procedures

On PND 3, male and female rat pups were grouped separately and were divided into four different subgroups on the basis of treatments–saline (Control), morphine (MOR), caffeine (CAFF), and morphine + caffeine (M+C) treatment. A total of 192 rat pups were used for the study. For western blot experiments: four animals per group (4 groups × 2 genders × 3 time-points = 96 animals (48 Male and 48 Female) and for immunofluorescence experiments: four animals per group (4 groups × 2 genders × 3 time-point = 96 animals (48 Male and 48 Female) were used. Animal care and experimental procedures were performed in accordance with the guidelines for animal care and use following approval by the Institutional Animal Care and Use Committee (IACUC, MWU file # 3076) of Midwestern University.

### Drugs

All drug doses were appropriately scaled keeping in mind the metabolic differences between rats and humans. All drugs were administered via a sterile filtered syringe with a 25-gauge needle. Each pup received treatments by subcutaneous injection with volumes adjusted as per body weight. Pups in control group were administered saline as per body weight with volumes ranging from 8 to 14 μl. The pups were recovered for 30 min following each injection in an incubator maintained at 34.5°C (nesting temperature) prior to being returned to their dams.

### Caffeine Administration

In clinical practice, caffeine is administered as a bolus of 20 mg/kg followed by daily maintenance doses of 5–10 mg/kg/day for days to weeks. This standard dosing regimen achieves 6–50 μg/mL of blood caffeine levels which is considered safe for premature infants (45). Similar levels are reached in a mice model with 100 mg/kg of caffeine citrate subcutaneously (46, 47). Pups in CAFF, M+C groups were administered 100 mg/kg of caffeine citrate (Fisher Scientific, Hanover Park, IL) on PND 3 (30) and repeated every 24 h for a total of 4 days (PND 3–6).

### Morphine Administration

In clinical practice, one method of administering morphine for acute pain is an intermittent bolus of 0.1 mg/kg morphine every 4 h intravenously. This dosing schedule attains a morphine level of 50–300 ng/ml in preterm infants to provide analgesic effects (48, 49). Comparable levels are closely achieved with a subcutaneous dose of 2 mg/kg/day in a neonatal rat pup model (50). Pups in MOR, M+C groups were administered 2 mg/kg of morphine sulfate (Henry Schein Animal Health, Dublin, OH, USA) on PND 3, repeated every 24 h for a total of 4 days (PND 3–6).

### Euthanasia

On PND 7, 14, and 28, 8 pups from each group of same gender were euthanized by decapitation, and the brains were removed for western blot and immunofluorescence analysis. The brain of each pup was weighed and stored at −80°C for western blot and 4% paraformaldehyde (PFA) for immunofluorescence analyses.

### Determination of CNS Proteins ET_A_, ET_B_, Drp1, Mfn2, Bcl-2, and Bax

#### Western Blot Analysis

Brain tissues were washed in chilled saline and homogenized in RIPA buffer (20 mM Tris-HCl pH 7.5, 120 mM NaCl, 1.0% Triton X-100, 0.1% SDS, 1% sodium deoxycholate, 10% glycerol, 1 mM EDTA, and 1 × protease inhibitor, Roche). Proteins were isolated in solubilized form and concentration was determined using Folin-Ciocalteu's Reagent. Solubilized protein (60 μg) was denatured in Laemmli sample buffer (Bio-Rad, Hercules, CA), resolved in 10% SDS–PAGE and transferred on nitrocellulose membrane (Sigma-Aldrich, St. Louis, MO, USA). The membrane was then blocked with superblock solution for 1 h at room temperature. The membranes were probed for anti-ET_B_, anti-ET_A_, anti-Drp1, anti-Mfn2, anti-Bcl-2, and anti-Bax (1:1000; Abcam, Cambridge, MA) primary antibodies overnight at 4°C. Membranes were then incubated with goat anti-rabbit and anti-mouse IgG, horseradish peroxidase-conjugated (HRP) secondary antibody (1:2000; Santa Cruz Biotech., Santa Cruz, CA, USA) for 2 h at room temperature. β-actin (1:10,000; Sigma-Aldrich, St. Louis, MO, USA) was used as a loading control. The chemiluminescence of HRP was visualized with SuperSignal WestPico Chemiluminescent Substrate (Thermo Fisher Scientific, Bartlett, IL) using the Kodak Gel Logic 1,500 Imaging System (CarestreamHealth Inc., New Haven, CT). The protein band intensity indicating the protein expression was analyzed using ImageJ (NIH) software and graphs were plotted after normalizing the protein expression with β-actin expression.

#### Immunofluorescent Analysis

To confirm western blot data, immunofluorescence technique was used to detect expression of Drp1, Mfn2, Bcl-2, and Bax markers in rat brain tissues. Rat brains were fixed in 50 ml of 4% paraformaldehyde (PFA) in NaPO4 buffer solution for 2 h at room temperature, and then submerged in 20% sucrose/4% PFA solution and stored at 4°C for 48 h. Brains were sliced into 20 μm thick slices using a cryostat (Microtome cryostat HM 505E; Walldorf, Germany) at −20°C. Tissue sections were washed three times with 1 × PBS and permeabilized with 1% Triton X-100 in PBS for 15 min at room temperature. Blocking with 5% BSA in 1 × PBS for 1 h at room temperature was carried out. The brain sections were incubated with anti-Drp1, Mfn2, Bcl-2, and Bax antibody (1:200 diluted in 1 × PBS) at 4°C overnight. Sections were washed twice in 1 × PBS and incubated with Alexa Fluor 488-conjugated donkey anti-mouse secondary antibody and Alexa Fluor 555-conjugated donkey anti-rabbit secondary antibody (1:200, Abcam, Cambridge, MA) for 1 h at room temperature in the dark and mounted with prolong gold anti-fade reagent with DAPI (Cell Signaling Technology, Danvers, MA, USA). Fluorescence was detected using an inverted fluorescent microscope (Nikon Eclipse TiE, Melville, NY). All images for analysis were taken using the same exposure with a multi-channel ND acquisition using NIS Elements BR imaging software (Nikon Instruments, Inc., Melville, NY). Analyses were performed using NIS-Elements 3.01 imaging software from Nikon Instruments, Inc. (Melville, NY).

### Assessment of Cell Damage

Cell damage, a measure of apoptosis, in brains of rat pups was evaluated using 7-amino actinomycin D (7AAD) assay on PND 7, 14, and 28. Rat pups brains were perfused with chilled 1 × PBS and fixed with 50 ml of 4% paraformaldehyde (PFA) solution for 2 h. Fixed brains were incubated in 20% sucrose/4% PFA solution, pH 7.4 at 4°C for 48 h. Brain tissue was sliced into 20 μm thick slices at −20°C using a cryostat (Microtome cryostat HM 505E; Walldorf, Germany). Tissue sections were blocked with 4% BSA in 1 × PBS for 30 min at 4°C. The brain sections were incubated with fluorescent DNA binding agent, 7AAD (0.25 μg/mL; Thermo Fisher Scientific, Bartlett, IL) for 15 min at 4°C. Sections were washed thrice in 1 × PBS and mounted with prolong gold anti-fade reagent with DAPI (Cell Signaling Technology, Danvers, MA, USA). Fluorescence was detected in a randomly fashion using inverted fluorescent microscope (Nikon Eclipse TiE, Melville, NY). Captured microscopic images were processed with NIS-Elements AR 4.13 software (Nikon, Melville, NY) and 7AAD positive nuclei were marked and counted. The data was represented as percent 7AAD positive cells normalized to the total number of nuclei in each field.

### Statistical Analysis

Power analysis was conducted using GraphPad Instat-3.1 with a beta of 0.8 and alpha of 0.05. The sample size in each group was *N* = 4 based upon expected change determined from results published in literature using similar procedures. Data are presented as mean ± S.E.M. One-way ANOVA followed by Bonferroni's *post hoc* comparison test was used. A *P* < 0.05 was considered to be significant. The statistical analysis was processed with GraphPad Prism 8.00 (GraphPad, San Diego, CA, USA). All groups were compared against control and one another.

## Results

### Effect of Treatment on Brain/Body Ratio

Brain/body weight ratio did not differ between different groups at different time points in both the genders.

### Effect of Treatment on Apoptotic Markers (Bax and Bcl-2)

**Figure 1:**
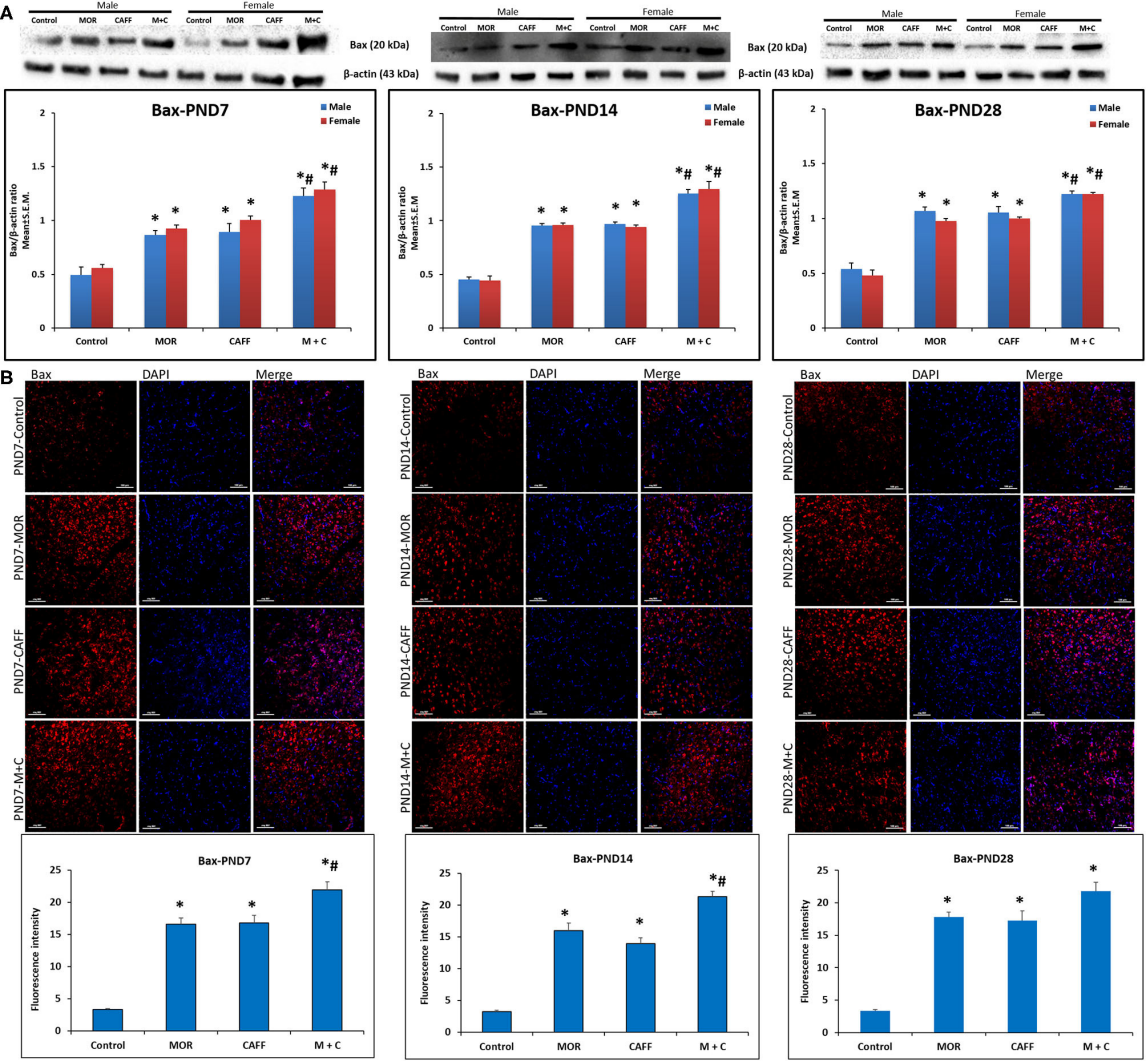
Expression of pro-apoptosis marker Bax in rat pups brain. **(A)** Western blots and densitometry graphs, **(B)** immunofluorescence images in Control, MOR, CAFF, and M+C treated pups brain tissues at PND 7, PND 14, and PND 28. **(A)** All blots are representative of four different experiments with similar results. β-Actin was used as a loading control. **(B)** Representative Immunofluorescence microscopy images and fluorescence intensity graphs of Bax. MOR, CAFF, and M+C groups marked increase in the expression of the pro-apoptosis marker Bax (red) compared to control. Nuclei were stained with DAPI (blue). Bar scale = 100 μm. Values are expressed as mean ± S.E.M. *P < 0.01 vs. Control, #*P* < 0.01 Vs. MOR and CAFF alone.

**Figure 2:**
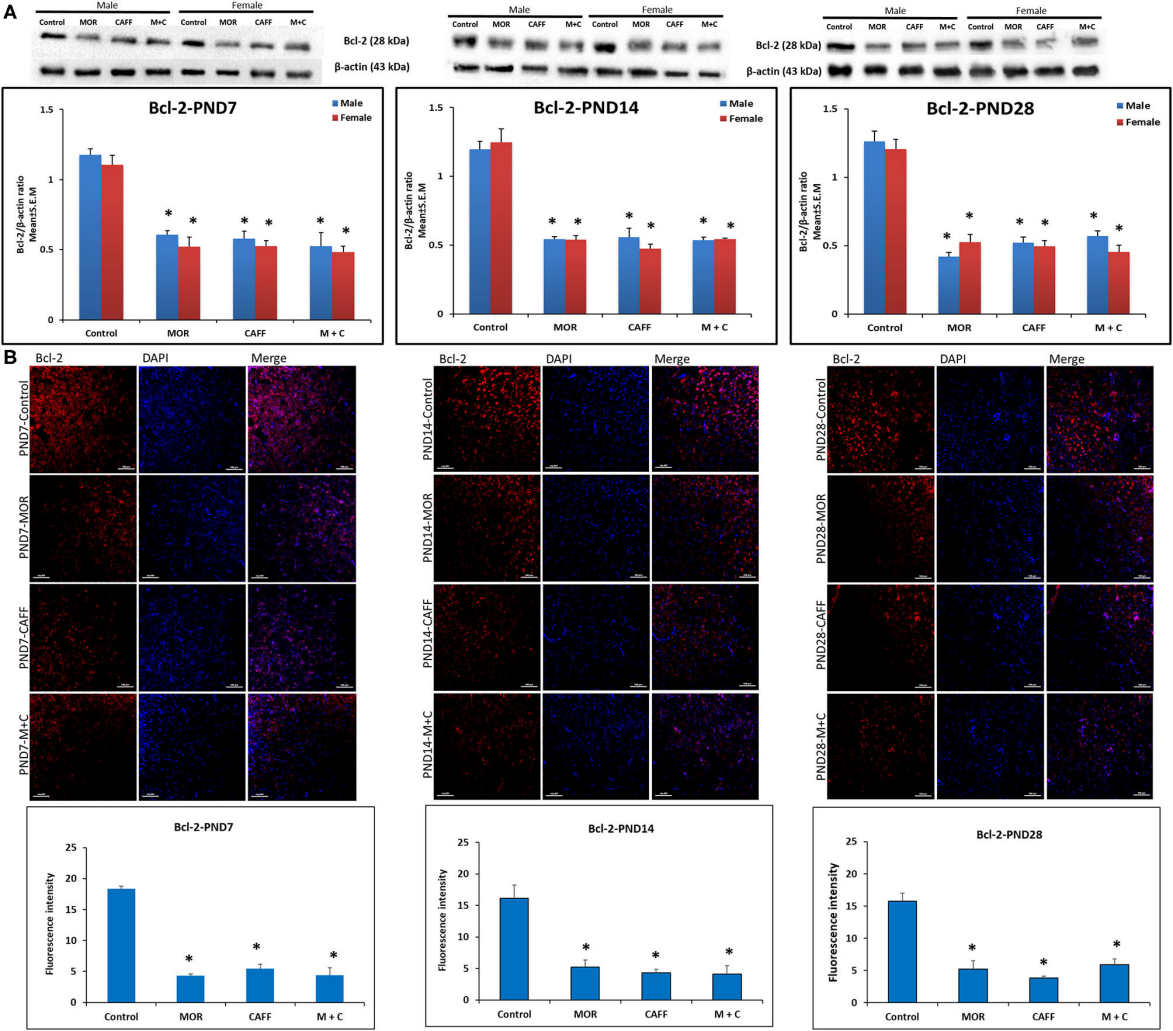
Expression of anti-apoptosis marker Bcl-2 in rat pups brain. **(A)** Western blots and densitometry graphs, **(B)** immunofluorescence images in Control, MOR, CAFF, and M+C treated pups brain tissues at PND 7, PND 14, and PND 28. **(A)** All blots are representative of four different experiments with similar results. β-Actin was used as a loading control. **(B)** Representative Immunofluorescence microscopy images and fluorescence intensity graphs of Bcl-2 markers. MOR, CAFF, and M+C groups marked increase in the expression of the anti-apoptosis marker Bcl-2 (red) compared to control. Nuclei were stained with DAPI (blue). Bar scale = 100 μm. Values are expressed as mean ± S.E.M. *P < 0.01 vs. Control.

Western blot analyses showed a significant (*p* < 0.01) increase expression of Bax in both male and female in MOR, CAFF, and M+C groups compared to control group at PND 7, 14, and 28 (Figure 1A). M+C had a markedly increased Bax expression compared MOR and CAFF alone at PND 7, 14, and 28 in female group and at PND 7 and 14 in male group (*p* < 0.01, Figure 1A). MOR, CAFF, and M+C groups had a significant (*p* < 0.01) decrease in Bcl-2 expression in both male and female groups compared to control group at PND 7, 14, and 28 (Figure 2A). Expression of Bcl-2 remained unaltered in the M+C group compared to MOR or CAFF in both male and female groups at different time points. No significant differences in the expression of these markers were noted between male and female subgroups. Therefore, these results were further confirmed with immunofluorescence imaging only in male group. The qualitative immunofluorescence data of these markers showed higher expression of Bax (red, Figure 1B) and lower expression of Bcl-2 (red, Figure 2B) in MOR, CAFF, and M+C treated groups compared to control.

### Effect of Treatment on Mitochondrial Function (Drp1 and Mfn2)

**Figure 3:**
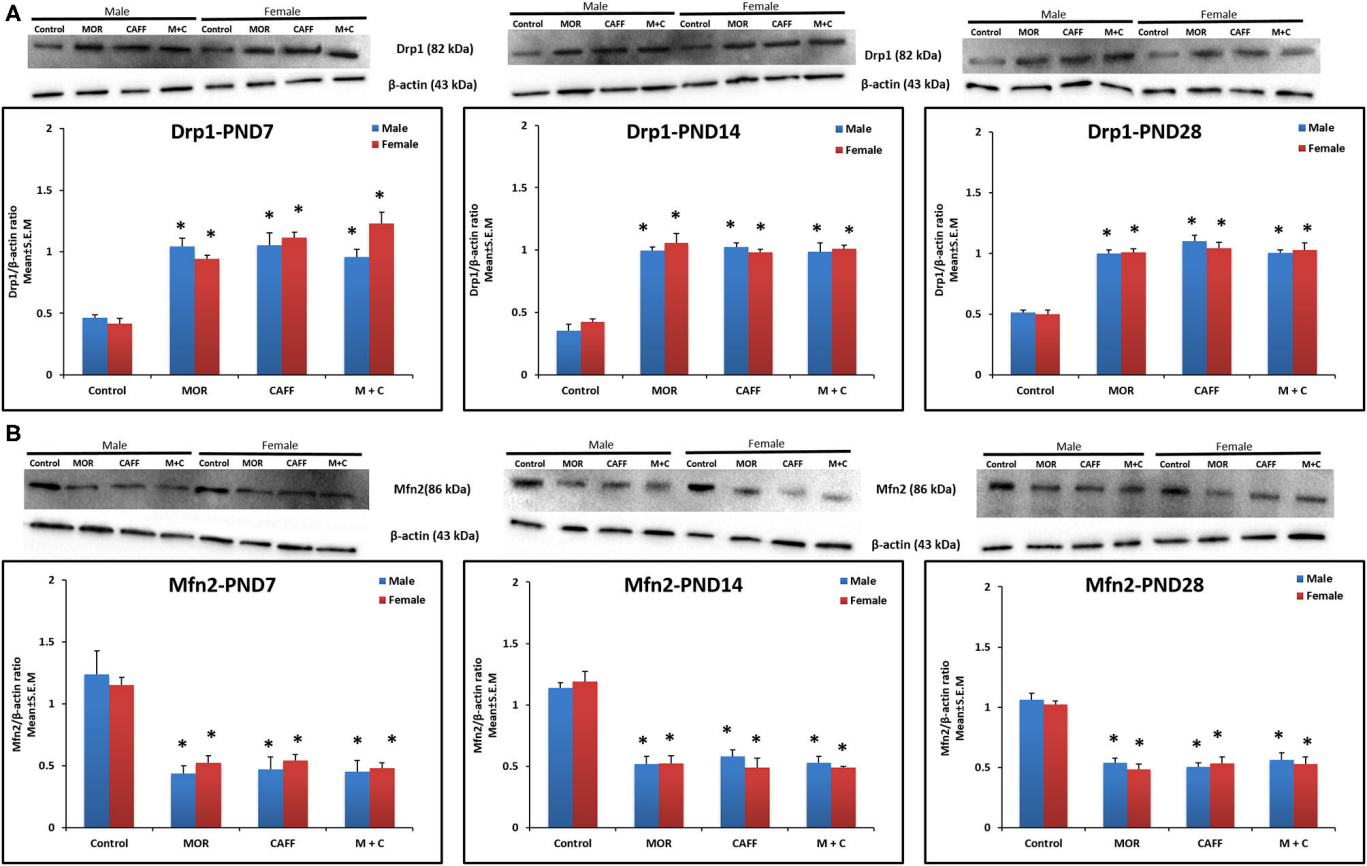
Expression of mitochondrial fission protein Drp1 **(A)** and mitochondrial fusion protein Mfn2 **(B)** in rat pup's brain. Western blots and densitometry graphs of Control, MOR, CAFF, and M+C treated pups brain tissues at PND 7, PND 14, and PND 28. All blots are representative of four different experiments with similar results. β-Actin was used as a loading control. Values are expressed as mean ± S.E.M. *P < 0.01 vs. Control.

MOR, CAFF, and M+C groups had a significant (*p* < 0.01) increase in Drp1 expression in both male and female groups compared to control group at PND 7, 14, and 28 (Figure 3A). MOR, CAFF, and M+C groups had a significant (*p* < 0.01) decrease in Mfn2 expression compared to control group in both male and female groups at PND 7, 14, and 28 (Figure 3B) in western blot analyses. Expression of Drp1 and Mfn2 remained unaltered in the M+C group compared to MOR or CAFF group in both male and female groups at different time points. No significant differences in the expression of these markers were noted between male and female groups. Therefore, these results were further confirmed with immunofluorescence imaging only in male group. The immunofluorescence data of these markers showed higher expression of Drp1 (green, Figure S1) and lower expression of Mfn2 (green, Figure S2) in MOR, CAFF, and M+C treated groups.

### Effect of Treatment on Cell Damage/Cell Death

**Figure 4:**
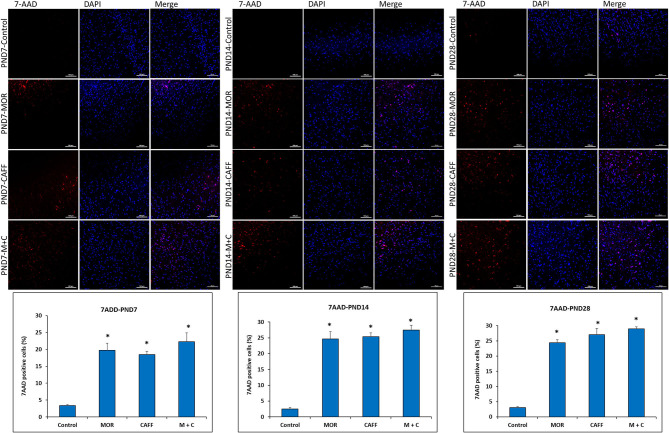
DNA Damage: Representative fluorescence microscopy images of brain tissue sections of Control, MOR, CAFF, and M+C treated pups at PND 7, PND 14, and PND 28 and a graph of quantified 7AAD^+^ cells. Scale bar = 100 μm. Values are expressed as mean ± S.E.M. *P < 0.001 vs. Control.

No significant differences in the expression of apoptotic and mitochondrial dysfunction markers were noted between male and female subgroups, therefore, we assessed the DNA damage only in male group. Detection of apoptotic cell nuclei, was done using fluorescent DNA binding agent, 7-amino actinomycin D (7AAD). 7AAD is a membrane impermeant dye that binds to double stranded DNA and exhibit dramatic increase in fluorescence intensity. Apoptosis in cells is known to cause damage to cell membrane, which allows 7AAD dye enter into the damaged cells. After staining with 7AAD, apoptotic cells show red fluorescence, while undamaged cells showed no fluorescence. No signals of cell death were observed in the control group at PND 7, 14, and 28 (3.3, 2.5, and 3.0% 7AAD+ cells, respectively). Treatment with MOR, CAFF, and M+C, significantly (*p* < 0.001) increased the number of apoptotic cells on PND 7 (19.7, 18.5, and 22.3% 7AAD+ cells, respectively), PND 14 (24.6, 25.4, and 27.4% 7AAD+ cells, respectively), and PND 28 (24.4, 27.0, and 28.9% 7AAD+ cells, respectively) (Figure 4).

### Effect of Treatment on Endothelin Receptors (ET_A_ and ET_B_)

Expression of ET_A_ and ET_B_ did not differ between different groups at different time points in either gender (Figure S3).

## Discussion

This is the first study aimed to examine how morphine in association with caffeine, affect mitochondrial function, expression of ET receptors and apoptotic markers in a neonatal rat model. The results provide some insight into the role of mitochondria in neuroapoptosis induced from the drug combination. An important consideration in the study design was to mimic a NICU practice where extremely preterm neonates are treated with morphine for pain/agitation (1) in association with caffeine for AOP. The study targeted the peak synaptogenesis time window of PND 3–6 in a rat pup, which corresponds to a premature neonate at 26–32 week gestation (51, 52).

The results demonstrate that concurrent exposure to clinically relevant doses of morphine and caffeine trigger cell death in an immature brain that persist at various developmental stages (PND 7, 14, and 28) suggesting the potential for long-term debilitating neurological outcomes. Findings also confirm the previously reported higher cell death mediated via mitochondria dependent caspase-3 activation from moderate dose of caffeine and morphine (30). This is further supported by other studies where the addition of caffeine to other sedatives such as midazolam/ketamine/fentanyl/alcohol in PND 3 rat pups has shown a supra-additive effect, causing more neurotoxicity than expected (28, 47).

In our study, morphine caused a significant increase in apoptosis via Bax, possibly by an increase in mitochondrial fission (via Drp1) and a significant decrease in mitochondrial fusion (via Mfn2) at PND 7, 14, and 28 in male and female pups. This may suggest a disturbance in mitochondrial function from an imbalance in fission/fusion proteins resulting in cell death. These results are in agreement with previous studies which have indicated that chronic morphine induces up-regulation of the Bax and down-regulation of the Bcl-2 protein (8, 53–55). Further, these studies show that mitochondrial viability plays a key role in the cell apoptosis (8, 53, 56). While previous studies involving acute low dose morphine did not find an increase in cell death, the discriminating factor may be partly explained by different dosage use and inhibition of apoptosis by activation of opioid receptors via other pathways like phosphatidylinositol 3-kinase/protein kinase B (PI3K/Akt) (19).

Similar to morphine, our data suggest that caffeine increased apoptosis via Bax possibly by an increase in mitochondrial fission (via Drp1) and a significant decrease in mitochondrial fusion (via Mfn2) at PND 7, 14, and 28 in male and female pups. This suggests a potential role of mitochondria in the cell death. Consistent with our results, previous studies have shown that caffeine has a neurotoxic effect on the immature brain (25, 30, 47). Additionally, we also assessed cell damage with 7AAD assay and observed significantly increased cell damage in morphine and caffeine treated animals.

The exact mechanism involved with neurotoxicity from M+C combination remains unknown. Caffeine stimulates the respiratory center in the CNS via adenosine receptor inhibition at therapeutic doses, but at higher doses causes cellular changes via phosphodiesterase inhibition, Gamma amino butyric acid A receptor (GABA_A_) inhibition and release of intracellular calcium (25, 57). On the other hand, morphine acts on the mu opioid receptor (MOR), the G protein coupled receptor, mediating pain and adverse effects, and at high concentrations can activate δ and κ receptors (58). At a cellular level, morphine inhibits adenylate cyclase decreasing cyclic AMP (Adenosine Monophosphate) and thereby calcium ion entry into the cell. In theory, considering the opposite effects of morphine and caffeine on the calcium ion homeostasis, there should be minimal effect on apoptosis from this combination. This dysregulation of calcium equilibrium can be a possible mechanism to trigger apoptosis by increase in pro-apoptotic mediator via Bax (40) and decrease in anti-apoptotic mediator via Bcl-2.

Several intrinsic pathways mediating apoptosis signaling act on the mitochondria leading to release of pro-apoptotic proteins. In our study, the M+C group had a significant increase in expression of Drp1 protein and a decrease in expression of Mfn2 protein, suggesting this imbalance in the fission/fusion processes may play a causal role in the neuroapoptosis induced from this combination. However, the expression of Drp1 and Mfn2 protein expression did not differ in the M+C group in comparison to caffeine, morphine groups, suggesting that caffeine did not augment the neurotoxicity when used in association with morphine via the mitochondrial processes.

Research has shown the involvement of endothelin (ET) in CNS development during critical stages of neuronal migration, organization, and myelination (32, 33). ET receptors also have extensive involvement in the analgesic actions of opioids such as morphine (59–62). Mitochondria are important to ET's role in the molecular regulation of neurogenesis and angiogenesis in cerebral ischemia (34). However, the role of endothelin receptor expression and its relation to mitochondrial function in neuroapoptosis induced from drugs in a neonatal rat model was never explored. In our study, the expression of ET_A_ and ET_B_ did not differ in the M+C group in comparison to other groups. It can be interpreted that administration of morphine and caffeine does not affect the expression of brain ETA receptors and ET_B_ receptors.

### Strengths of our Study

This is a pilot study to highlight the effects of morphine and caffeine, two most commonly used drugs in the NICU on central nervous system proteins. The study also provides evidence for the possible mitochondrial mechanism underlying morphine and caffeine induced neuronal damage. In future, this study will help in exploring the potential protective effect of selective Drp1 inhibitors in neurotoxicity induced from drug combinations routinely used in the NICU. Caution should be exerted when using caffeine with morphine in the NICU.

Our study has some important limitations: First, our neonatal rat model was used to model clinical practice of morphine and caffeine exposure in the absence of pain or other stress encountered in the NICU. While, some studies reported a model of neonatal stress and morphine treatment which produces long lasting neurobehavioral effects in adult rats (16), others showed that low doses of morphine may protect the brain from neurodegeneration in the presence of pain (63). Second, animal brain development is similar to humans, however, translating the findings from animal studies to humans is difficult and therefore, clinical studies are needed to address the long-term implications of morphine in association with caffeine in the preterm infants. Third, unlike previous studies, which noted specific regional brain damage based on receptor location, our study utilized the whole brain homogenates opposed to specific regions for CNS protein expression. A prior study has shown that caffeine in association with morphine caused greater cell death in thalamus than either drug alone at 24 h after drug administration (30). Further studies exploring thalamic areas for cell death at PND 7, 14 and 28 would be interesting. Additional studies looking into Drp1/Mfn2 localization and translocation from cytosol to mitochondria would be valuable.

In summary, we found that early exposure to morphine and caffeine alone and in association significantly induced apoptosis and caused disturbance in mitochondrial fission and fusion proteins suggesting involvement of mitochondrial dysfunction in apoptosis. Early exposure to morphine and caffeine combination induced greater apoptosis compared to morphine and caffeine alone. However, this combination did not demonstrate significant mitochondrial dysfunction compared to morphine and caffeine alone. Further molecular studies exploring other mechanisms of neurotoxicity may play a role in developing potential neuroprotective agents to minimize the neurotoxicity.

## Data Availability Statement

The raw data supporting the conclusions of this article will be made available by the authors, without undue reservation.

## Ethics Statement

The animal study was reviewed and approved by Institutional Animal Care and Use Committee (IACUC, MWU file # 3076) of Midwestern University.

## Author Contributions

SK Development of hypotheses, research, and grant proposal. Performed laboratory experiments, data analysis, and interpretation. PP Research mentor and advisor. GS Research mentor and advisor. SB Oversight and carried out laboratory experiments, data generation, analysis, and interpretation of results. AR Standardization of 7AAD assay and data analysis for cell death assay. RD Guidance with data analysis and manuscript preparation. AG Oversight of basic science laboratory at Midwestern University. All authors contributed to the article and approved the submitted version.

## Conflict of Interest

AG is founder and CEO of company Pharmazz Inc. The remaining authors declare that the research was conducted in the absence of any commercial or financial relationships that could be construed as a potential conflict of interest.

## Figures and Tables

**Table:** 

Mitochondrial fission protein (Drp1)
Mitochondrial fusion protein (Mfn2)
Postnatal day (PND)
Bcl-2 associated x protein (BAX)
Endothelin A receptor (ET_A_)
Endothelin B receptor (ET_B_)
Neonatal Intensive Care Unit (NICU)
Central nervous system (CNS)
Apnea of Prematurity (AOP)
Caffeine for apnea of prematurity (CAP)
Bronchopulmonary dysplasia (BPD)
Endothelin (ET)
Institutional Animal Care and Use Committee (IACUC)
Midwestern University (MWU)
Paraformaldehyde (PFA)
Phosphate-buffered saline (PBS)
Bovine Serum Albumin (BSA)
Phosphatidylinositol 3-kinase (PI3K)
Protein kinase B (Akt)
Gamma amino butyric acid receptor (GABA_A_)
7-amino actinomycin D (7AAD)
