# Supplementary figures and images for: Exposure to Morphine and Caffeine Induces Apoptosis and Mitochondrial Dysfunction in a Neonatal Rat Brain
Source: Front Pediatr. 2020 Sep 18;8:593. doi: 10.3389/fped.2020.00593 (PMC7530195; doi:10.3389/fped.2020.00593)

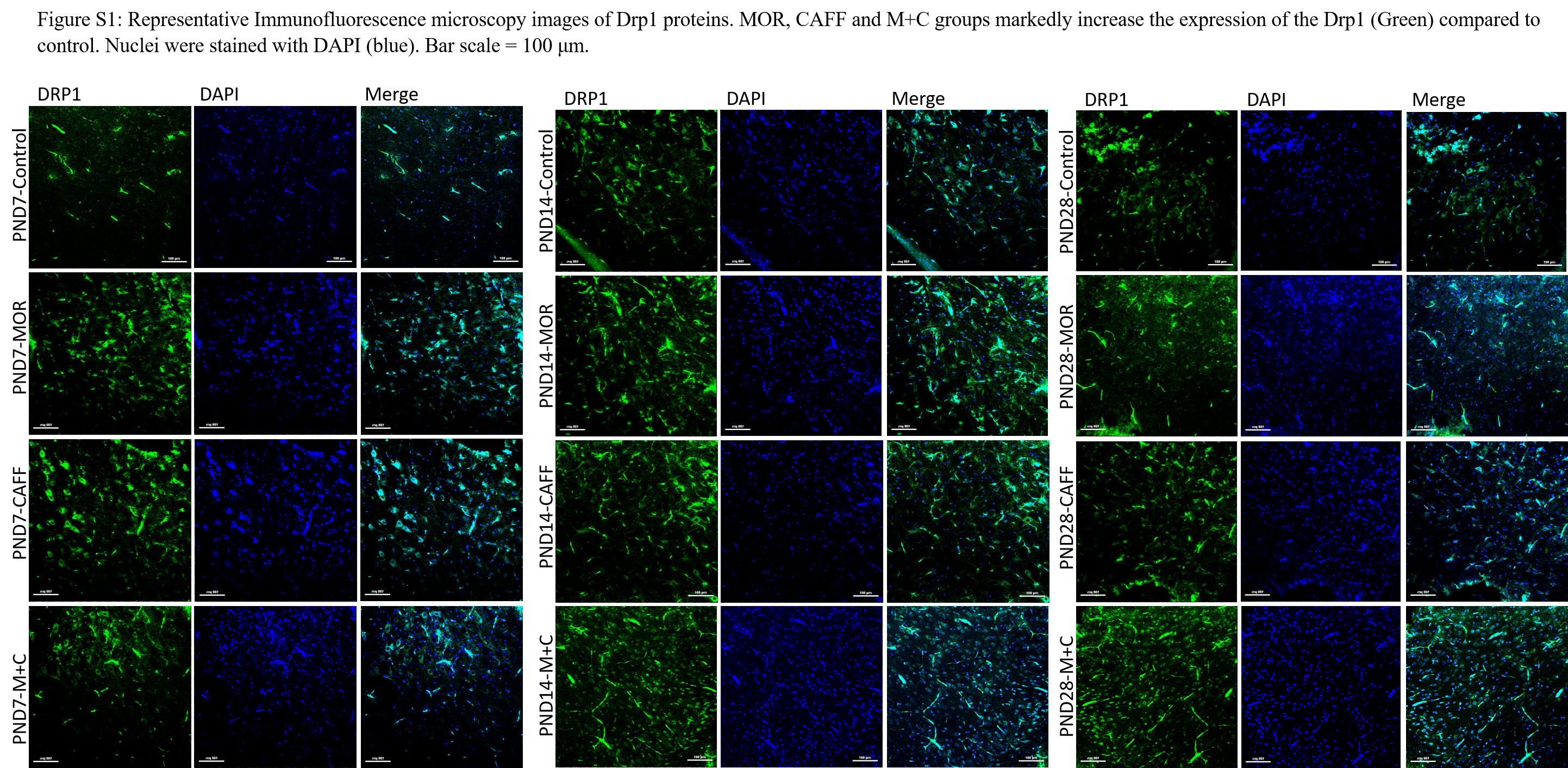

Supplement: Supplementary file 1 [file Image_1.TIF]

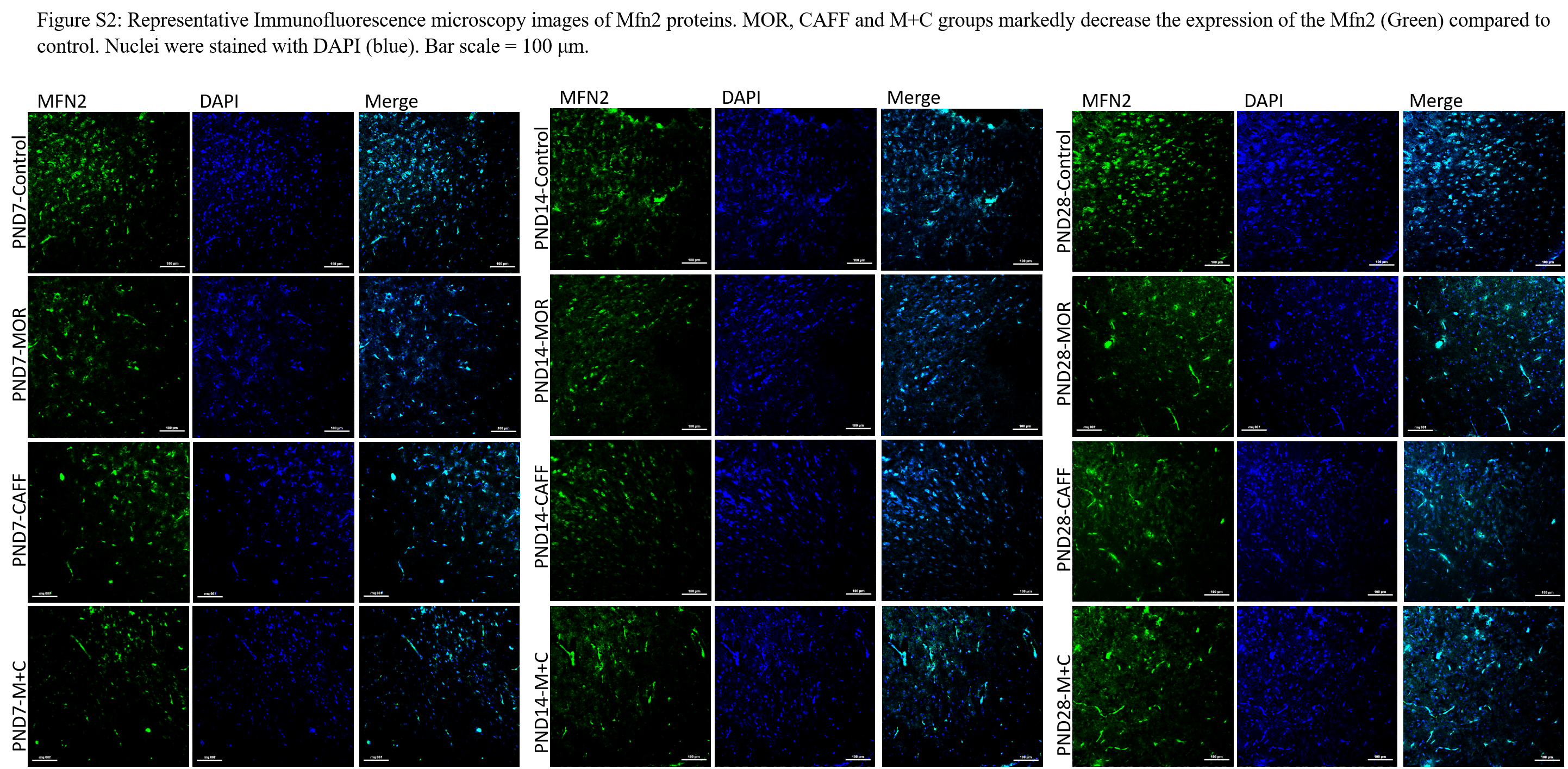

Supplement: Supplementary file 2 [file Image_2.TIF]

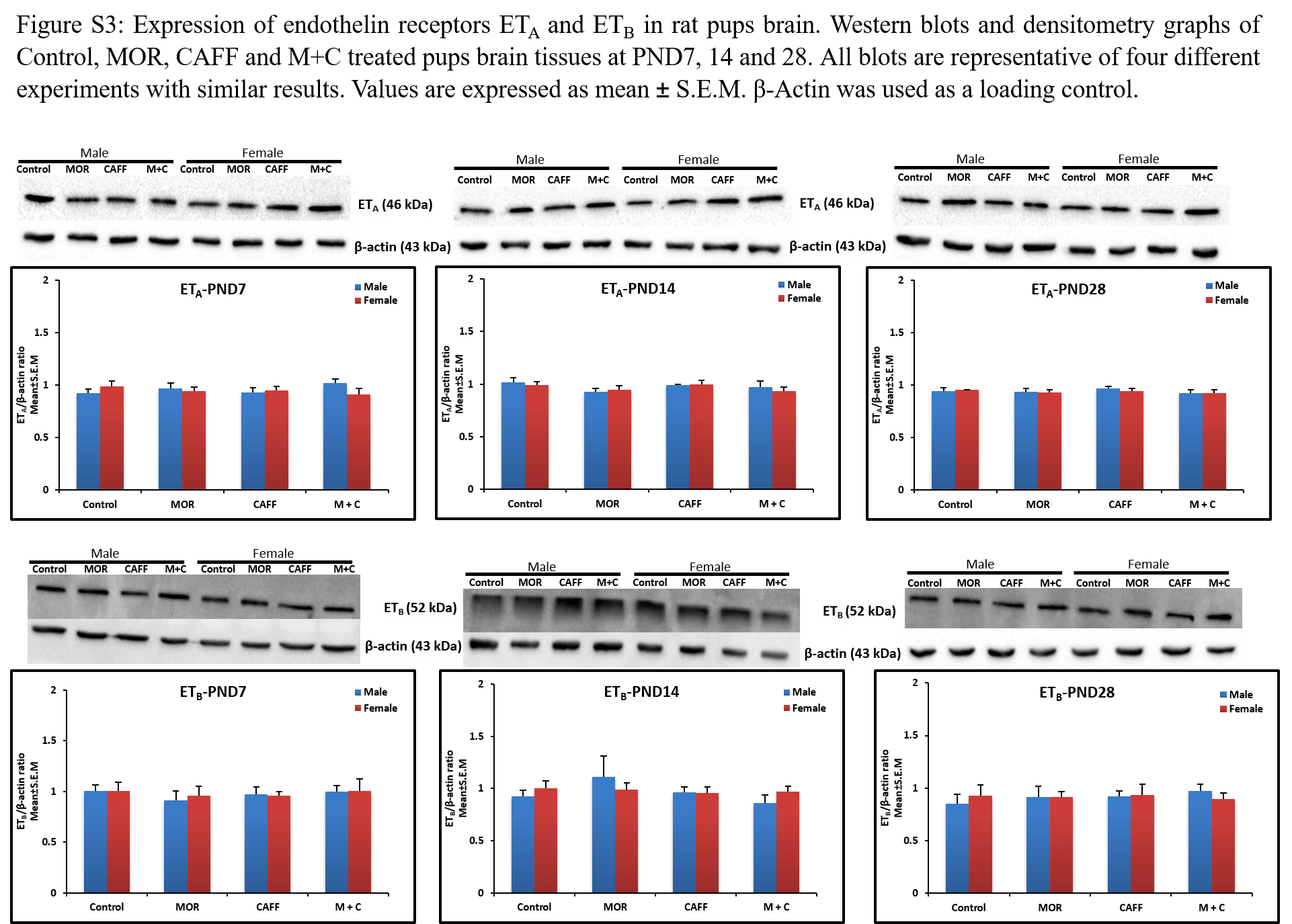

Supplement: Supplementary file 3 [file Image_3.TIF]
